# Data visualization as an intervention for pediatric chronic pain: a pilot feasibility study protocol for a randomized controlled crossover trial

**DOI:** 10.1186/s40814-022-01170-5

**Published:** 2022-10-03

**Authors:** Katelynn E. Boerner, Unma Desai, Karon E. MacLean, Tamara Munzner, Haley Foladare, Javed Gill, Tim F. Oberlander

**Affiliations:** 1 Department of Pediatrics, University of British Columbia, Vancouver, BC Canada; 2 BC Children’s Hospital and Research Institute, Vancouver, BC Canada; 3 Department of Computer Science, University of British Columbia, Vancouver, BC Canada; 4 Department of Cognitive Systems, University of British Columbia, Vancouver, BC Canada; 5 Patient partner, Vancouver, BC Canada; 6 School of Population and Public Health, University of British Columbia, Vancouver, BC Canada

**Keywords:** Chronic pain, Children and adolescents, Data visualization

## Abstract

**Background:**

Chronic pain is a common and costly condition in youth, associated with negative implications that reach far beyond the pain experience itself (e.g., interference with recreational, social, and academic activities, mental health sequelae). As a self-appraised condition, pain experience is influenced by patient’s biases and meaning-making in relation to their symptoms and triggers. We propose that interacting with self-reported data will impact the experience of pain by altering understanding and expectations of symptom experience and how pain interacts with other factors (e.g., sleep, emotions, social interactions). In this study, we aim to establish the feasibility and acceptability of using a data visualization platform to track and monitor symptoms and their relationship with other factors, versus simply daily reporting of symptoms using a smartphone-based Ecological Momentary Assessment (EMA).

**Methods:**

This protocol is for a randomized, single-center, open-label crossover trial. We aim to recruit 50 typically developing youth aged 12–18 years with chronic pain to take part in two phases of data collection. The trial will utilize an A-B counterbalanced design in which participants will be randomly assigned to receive either Part A (EMA alone for 7 days) or Part B (EMA plus visualization platform for 7 days) first and then receive the opposite phase after a 7-day break (washout period). Key outcomes will be participant reports of acceptability and feasibility, EMA completion rates, barriers, and perceptions of the benefits or risks of participation. Secondary exploratory analyses will examine the relationship between EMA-reported symptoms over time and in relation to baseline measures, as well as pilot data on any improvements in symptoms related to engaging with the data visualization platform.

**Discussion:**

This protocol describes the feasibility and pilot testing of a novel approach to promoting self-management and facilitating symptom appraisal using visualized data. We aim to determine whether there is a sufficient rationale, both from the perspective of feasibility and patient satisfaction/acceptability, to conduct a larger randomized controlled trial of this intervention. This intervention has the potential to support clinical care for youth with chronic pain and other conditions where self-appraisal and understanding of symptom patterns are a critical component of functional recovery.

**Trial registration:**

Open Science Framework doi: 10.17605/OSF.IO/HQX7C. Registered on October 25, 2021, osf.io/hqx7c

**Supplementary Information:**

The online version contains supplementary material available at 10.1186/s40814-022-01170-5.

## Introduction

### Background and rationale

Chronic pain is a common, costly condition in youth, of whom 3–5% report disabling levels of chronic pain [1]. Even more youth (20–30%) report recurrent pain that is not disabling, but still interferes with academic, social, and recreational functioning, and that has significant effects on mental health (e.g., higher rates of anxiety, depression, and post-traumatic stress symptoms) and quality of life [2–5]. As a self-appraised condition, pain is whatever the patient says it is. Self-appraisals can be biased by our attention, interpretations, and memory. We posit that interacting with lived-experience data—through viewing and reflecting on daily patterns—will help patients understand and communicate their pain experience and the factors that influence it, enabling a process of active engagement leading to pain relief, thereby making “the data the drug”.

Ecological momentary assessment (EMA) is used to obtain in-the-moment longitudinal assessments of feelings or behavior in the context of daily social life [6, 7] and enhance psychological interventions [8, 9]. EMA methodology has been frequently used in adult chronic pain research [10], but has been underused in pediatrics. To date, the majority of the literature on the use of EMA (or similar methods, such as daily diaries) in pediatric chronic pain has focused on specific disease populations (e.g., sickle cell disease, cancer, arthritis, headache) and the EMA has primarily measured features of the pain (e.g., intensity, location), sleep, functioning, and medication use [11–17]. EMA can be used to quantify changes in anxiety over time [18, 19], and the relationship between emotions and contextual factors such as peer interactions [7] in a typical everyday environment. The use of youth’s smartphones as a data collection tool allows for more ecologically valid sampling that avoids problems related to retrospective reporting [20] and makes participation more accessible to youth who live in rural and remote areas. This approach harnesses a technology that is already readily used by youth; Statistics Canada reported that in 2018, 97.9% of Internet users aged 15–24 years had a smartphone, and 57.5% checked their smartphone at least every 30 min [21].

The use of EMA also allows for data collection unconstrained by the original constructs of established questionnaires (i.e., anxiety questionnaires will assess the perception of anxiety in one specific timeframe/context). This data offers a unique opportunity to examine temporal relationships between symptoms and experiences that have not been previously investigated in youth with chronic pain. Importantly, while the literature on the use of EMA in pain research is well-established, it is generally described as a one-way data collection tool, whereby participants submit their diary ratings but are not provided any specific feedback or opportunities to interact with their data [22–24]. In cases where a data summary report is shared back with participants, this is conceptualized as more of an assessment outcome than an active intervention, whereby participants are provided a personalized profile at the end of the study but do not interact with their data throughout the data collection period [25].

We hypothesize that allowing pediatric patients to visualize personal health data in an accessible and youth-focused format will enable them to validate their perceptions around their chronic pain journey, understand how their symptoms relate to other areas of their everyday lives, and track and improve their general functioning. We propose building an interactive and personalized data visualization mobile application which will allow clinicians and patients to view EMA-collected data in ways which may aid in their treatment or pain-management process [26]. Such an approach to data collection may provide the opportunity for mutual benefit to all key user groups, namely researchers, clinicians, patients, and their families. We have focused our daily data collection on variables that are known to affect the functioning and treatment outcomes of youth with chronic pain, but where there is not compelling data for how these variables fluctuate and interact with pain on a day-to-day basis; namely, emotions, somatic symptoms, social experience, and sleep [15, 27–30].

Applying digital health approaches to the treatment of chronic pain is not new. A 2015 study identified nearly 300 pain self-management apps, but found they were overly simplistic, lacked involvement of health professionals or theoretical basis, and had not been rigorously tested for effectiveness [31]. The present approach is based on the Common Sense Model of Self-Regulation by Leventhal and colleagues [32], where youth act as “common-sense scientists” by populating and reflecting on informative representations of their symptoms, generating goals for self-management, and receiving feedback. This approach is consistent with evidence-based strategies for pain management (i.e., CBT, cognitive behavioral therapy), whereby patients are asked to notice patterns between thoughts, feelings, and behaviours. The digital application designed for this protocol takes input from health professionals, computer science and visualization experts, young people, and other relevant stakeholders. It overcomes a major therapeutic barrier: obtaining reliable, understandably portrayed data from patients’ everyday life upon which to evaluate these patterns and set goals.

### Objectives

We will determine whether visualization of multiple dimensions of real-world self-reported measures of somatic symptoms, emotions, functioning, and social experience is usable, accessible, and understandable to youth with chronic pain. We will also collect preliminary data on the relationship between daily reported emotions and somatic symptoms, social experience (peer interaction), and functioning (sleep, school attendance) in youth with chronic pain, as assessed by EMA self-report, as well as preliminary data on whether interacting with visualized data of these interactions can be effective in supporting youth to understand and manage their pain and associated symptoms. This pilot feasibility study is intended to be the first step in conducting a larger randomized trial of data visualization as an intervention for chronic pain in the future. This project is designed to put patients at the center of a meaningful data supply-review-act-iterate process. We envision enabling participants to use their own real-world data to make decisions about how to manage their pain [33]. To our knowledge, this is the first project to harness the visualization of youth’s symptom-tracking data as the intervention itself.

### Materials

#### Design and development of personal data visualization intervention

The initial development of the visualizations was conducted as part of a graduate design course team project offered by the University of British Columbia’s *Designing For People (DFP)* graduate training program. Pilot EMA data was collected from the five graduate students working on the project, and prototype visualizations developed with input from content experts and youth. Our initial data visualization dashboard was developed with a diverse group of students and faculty, representing a variety of racial, ethnic, cultural, and language backgrounds with a common goal of making data understandable to a wide audience. The current version of the “personal data visualization dashboard” offers multiple customized approaches (using standard charts like line charts, bubble charts, bar graphs, heatmaps, as well as custom-designed visualizations) to visualizing dimensions such as sleep, emotional well-being, physical health, and social interactions based on the individuals’ own EMA data. The primary objective of the visualizations is to reveal how these dimensions are interacting with each other for this individual (e.g., how sleep influences anxiety). These visualizations have been developed in an iterative process, considering primary patient and clinician requirements. The design and prototyping of these visualizations were guided by information visualization and human-centered design experts and validated with clinician feedback. It implements user-centered design and data visualization principles, to ensure an optimal patient-centered application. Industry partners *Careteam* [34] will provide their clinically tested and broadly available platform to capture the baseline questionnaires, record daily EMA data, and present our data visualizations. Further development in collaboration with *CareTeam* and pilot-testing engaging youth with chronic pain is currently underway and will be described in a forthcoming publication. The visualizations with their patient-specific data will be embedded directly in the CareTeam web platform for real-time results and ease of use. A near-final draft of the sample visualizations (currently undergoing user testing) is available in Additional file 1.

## Methods

This protocol was prepared in accordance with the SPIRIT (Standard Protocol Items: Recommendations for Interventional Trials) 2013 guidelines for reporting protocols of clinical trials [35, 36] and the CONSORT (Consolidated Standards of Reporting Trials) extension for pilot and feasibility trials [37]. A SPIRIT checklist has been included as Additional file 2.

### Study design

We will conduct a randomized, single-center, open-label crossover trial with a 1:1 allocation ratio, exploratory framework.

### Setting

This study will be conducted out of the Complex Pain Service (CPS) at a large tertiary-care hospital in Western Canada. The CPS provides assessment, consultation, and treatment of chronic, complex, and/or persistent non-cancer pain for children and youth up to age 18. Children and youth are referred to for wide range of painful conditions where, regardless of their etiology, pain commonly significantly interferes with daily function and has persisted despite treatments implemented by the community/referring provider.

### Participants

We will aim to recruit 50 youth with complex pain from the CPS.

#### Inclusion criteria

Youth will be eligible if they are between 12 and 18 years old and have any type of chronic pain (i.e., pain that has persisted for >3 months). Youth and parents must have sufficient command of English to provide consent/assent and for the youth to complete the study tasks in English. Youths are not required to have a smartphone to participate.

#### Exclusion criteria

Youth are not eligible if they have a diagnosis of cerebral palsy, autism spectrum disorder, learning disabilities, attention-deficit-hyperactivity disorder, or a genetic/metabolic disorder that interferes with their ability to complete the tasks required for this study (i.e., self-report on their symptoms and provide feedback on their experience). Of note, our team is undergoing separate research to investigate the use of in-home data collection and visualization in youth across the developmental spectrum [38].

#### Recruitment

Potential participants will be contacted by email by a Research Coordinator (not involved in their clinical care) and invited to complete the study. Participants will be recruited from active/recent patients in the Complex Pain Service and from past participants of research studies conducted in our clinic who consented to be contacted about research opportunities^1^. Our recent research studies have had a 90.5% rate of consent to contact regarding research opportunities, and a 62% enrollment rate in research studies (with a 23% drop-out rate among those who consented to participate) in available studies from this same population [38]. If needed, we intend to use purposive sampling to recruit a sample of participants for the present project that reflects the diversity observed in the CPS.

### Procedures

**Fig. 1:**
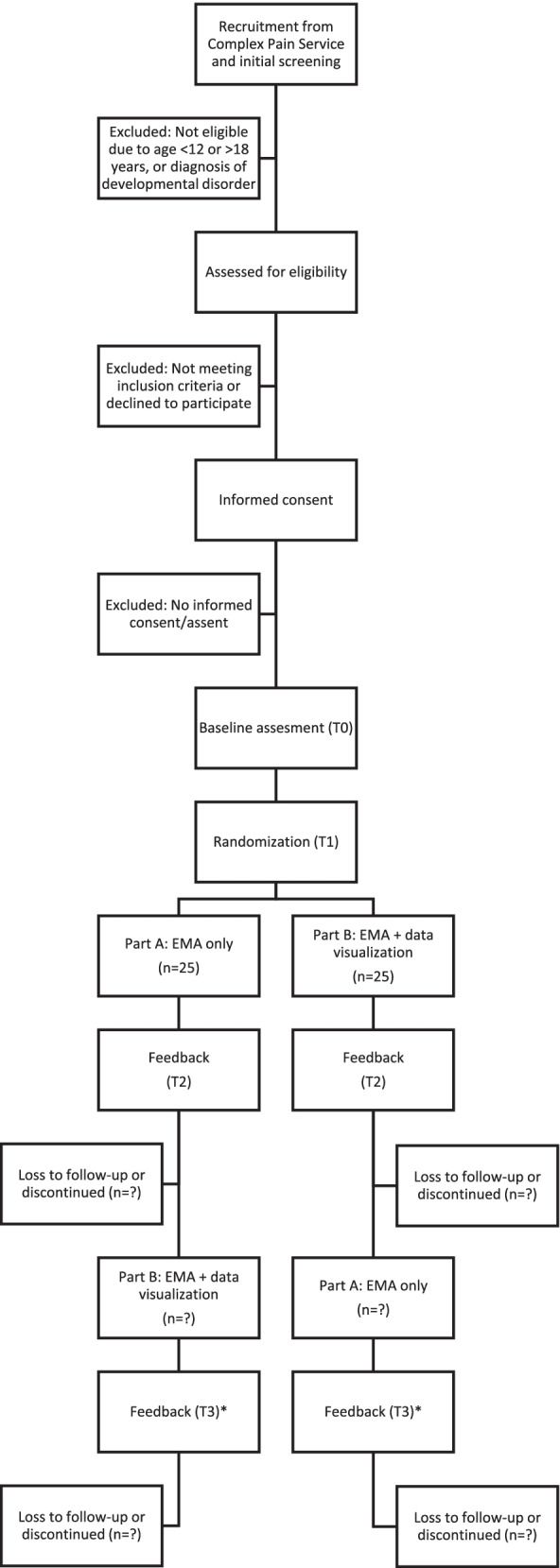
Flow chart of study participation. *A subset of participants who complete T3 feedback will be invited to complete an in-depth interview and questionnaires regarding the visualizations following trial completion

Complete procedures are outlined in Fig. 1. Consent and assent for all participants will be obtained from youth and parents/caregivers. Youth will complete baseline questionnaires prior to randomization. Once participants have been assigned to groups, the smartphone-administered EMA data collection will be completed by the youth for two 7-day periods with a 7-day break in between (washout period). Participants who do not have access to a smartphone will be loaned a pre-paid, data-enabled smartphone for the duration of the study.^2^ The trial will utilize an A-B crossover design in which participants will be randomly assigned to receive either Part A (EMA alone) or Part B (EMA plus visualization) first and then receive the opposite phase after the 7-day break. In Part A, the data collection will occur through EMA (repeated self-report via smartphone). In Part B, the data visualization will appear immediately after data entry, updating as new data is added and allowing the participant to interact with their data as the visualizations change throughout the week. Youth will be oriented with basic instructions on how to navigate the visualization platform, what the visualizations represent, and how to interpret them.

EMA will consist of repeated self-report measures of emotions and somatic symptoms, social experience such as interactions with peers, avoidance, context (e.g., family, friends, home, school attendance), and sleep duration/quality. Questions include a mix of visual analogue scales, free response, and interval or nominal checkboxes. Participants will complete the EMA for 7 consecutive days (3 prompts/day, at morning, afternoon, and evening using a fixed time-based sampling method employing a coverage model (e.g., asking about the time since the last prompt)) using the *CareTeam* platform, developed and deployed by our industry partner [34]. EMA procedures will be set up using established best practices for use in children and adolescents [39, 40] and previously used measures in a pediatric anxiety sample [7]. The development of the EMA platform was pilot tested with youth, trainees, and investigators, and integrated preliminary feedback from a concurrent initiative we are developing that is soliciting feedback from patients and families regarding the development of an in-home data collection platform [38].

After completion of each phase, youth will be asked to evaluate their experience with the EMA (and, at the end of Phase B, the data visualization) with an investigator-created questionnaire; see Additional file 3. A subset of youth will be invited to take part in an interview at the end of the trial to provide more in-depth feedback on the visualization platform.

#### Informed consent

Consent (from parent/legal guardian) and assent (from youth) will be obtained. Potential participants will be contacted by email by a researcher (not involved in their clinical care), introducing the study with an invitation letter and consent form, and providing the opportunity for the family to opt out. The researcher will follow up a week later to see if the family is interested in participating. If so, the researcher will review the consent form with the parent and youth over the phone and send them the link to complete the e-consent forms using REDCap, a secure online system hosted by the BC Children’s Hospital Research Institute [41].

#### Confidentiality

All research data will be identified using a specific code number assigned to each participant. Any personally identifying information (e.g., name, date of birth) will not be included on any research record that has the participant’s code number. The master list of participants and their corresponding codes will be kept in a password-protected file on a secure server and will have limited access. No information with personal identifiers will be released to outside agencies during or after the study without written consent from participants. Any phones borrowed by study participants will be restored to factory settings upon return to eliminate any possibility of the identity of participants becoming known to others that may use the phone.

Only those directly involved in the study will have access to study data, including the Principal Investigator, Co-Investigators, and any research assistants or trainees added to the project. All e-consent/assent forms and questionnaires will be collected using the secure, web-based application, Research Electronic Data Capture (REDCap), via the BC Children’s Hospital Research Institute Clinical Research Support Unit. *CareTeam* [34], which is the platform that will be hosting the EMA questions and visualizations, accesses aggregate data in an ongoing capacity for metrics and only access individual data if providing support to a specific user for a technical problem.

#### Baseline assessment

The following domains will be assessed prior to randomization at the baseline period:

- Demographics and contextual factors: Youth will self-report their assigned sex and gender identity [42], health, and medication/treatment use. Youth will report on pandemic-related stressors using the *COVID-19 Impact Questionnaire* (adapted by Kopala-Sibley, Noel & Birnie at the University of Calgary, from impact measures of natural disasters on mental health [43], which assesses financial, health, social, occupational, and academic impacts of the pandemic. This measure does not contain items that directly overlap with mental illness to prevent inflated associations with anxiety measures.
- Pain characteristics and interference: Youth will provide reports of pain duration, pain intensity, and interference (using the *PROMIS* (Patient-Reported Outcomes Measurement Information System ®) pain scales [44, 45]).
- Anxiety and depression symptoms: Youth will provide reports of symptoms of anxiety and depression using the *PROMIS* Emotional Distress scales [46, 47].
- Somatic symptoms: Reports of youth’s somatic symptoms will be provided by youth using the well-validated *Children’s Somatic Symptoms Inventory-8 item version* [48]*.*

### Randomization

#### Sequence generation

The sequence of randomization to assign participants to start with either Part A (EMA) or Part B (EMA + data visualization) will be generated by the investigators using computer-generated random numbers with a 1:1 allocation ratio. No blocking or stratification will be used.

#### Concealment mechanism

Allocation sequence will be implemented using sequentially numbered, opaque, sealed envelopes.

#### Implementation

A research assistant, who was not involved in the sequence generation and does not have access to the randomization list, will enrol participants and assign them to interventions based on the next assigned envelope.

#### Blinding

Data analysts will be blinded to participant allocation. The only exception to this will be the qualitative analysis of the questions regarding satisfaction, etc., of the visualization platform, as this question will only be asked after participants complete Part B. Due to the nature of the study, participants and care providers will not be blinded.

### Interventions

#### Explanation for the choice of comparators

Comparing the EMA + visualization to EMA alone will allow us to determine the effect of self-monitoring by collecting data alone compared to the effect of “seeing” visualized data.

#### Intervention description

The intervention is described in detail in the “Materials” section above. Briefly, the intervention will involve access to the data visualization dashboard, where participants can view visualizations of the data they are inputting in the EMA to observe trends and interactions of different dimensions over time.

#### Criteria for discontinuing or modifying allocated interventions

Discontinuation of any stage of the trial for a particular participant will occur if the participant requests as such and/or if the participant, parent/legal guardian, researcher, or clinician deems that the intervention or participation in the trial is causing harm (e.g., emotional distress). Should this occur, the case will be discussed with the Data Monitoring Committee (DMC), who will determine whether there is sufficient concern to halt the study entirely.

#### Strategies to improve adherence to interventions and to promote participant retention

The compensation structure is set to $20 at baseline, with an additional $10 for completing each stage of EMA data collection with at least two thirds (i.e., 14 of 21) of the data points completed, which is designed to encourage data completeness and reduce attrition, in accordance with published recommendations for conducting EMA studies with youth [39]. Adherence will be monitored by examining rates of EMA completion, self-reported use of the data visualization platform, and data analytics of usage of the data visualization platform (anonymized and in aggregate). For participants who discontinue their participation, we will record the reason why.

#### Relevant concomitant care permitted or prohibited during the trial 

Participants will be encouraged to continue to engage in any other treatments for their chronic pain concurrently with trial participation. As the 3P approach to chronic pain management (combination of physical, psychological, and pharmacological therapies) is the standard of care, it was concluded to be unethical to stop or delay any of these treatments during the trial. Indeed, the intention of the data visualization intervention would be to supplement or support other therapies; therefore, the concurrent use of additional interventions was deemed to be the most ecologically valid and ethical approach. Participants will be asked to report the use of any concurrent interventions during the trial at baseline and during each feedback questionnaire.

Two additional measures have been put in place to provide support to participants during the trial. Resources for pain and mental health care are included on the consent and assent forms for participants to reference. Additionally, during EMA assessment periods, the participant will receive a prompt at the end of each day to try a free self-management application (*Rootd*; www.rootd.io). *Rootd*’s content is based on cognitive behavioral therapy and includes lessons on understanding and managing anxiety in the short and long term, a journal tool, exercises, and step-by-step guides to strategies such as deep breathing and active visualization. The app also features an emergency contact button to call a loved one or hotline if in distress and a Personal Stats Page to track user progress.

#### Provisions for post-trial care

No specific provisions have been made for post-trial care; participants will be encouraged to continue with their current treatment plan as directed by their care providers. However, should a participant express emotional distress in response to trial participation, either during or after the trial, they will be provided with mental health resources and referral suggestions, if appropriate (see the section below on adverse events).

### Outcomes

#### Primary feasibility outcomes

Feasibility will be established through the following measures: (a) recruitment rate (including reasons for declining participation, with a particular focus on equity barriers related to internet/data access, smartphone access), (b) retention rate (i.e., number of patients that complete the entire trial), (c) data completion rate (i.e., the number of EMA data points completed by the participants during the trial) and timeliness/duration of completion (i.e., were EMA ratings completed in the appropriate time frame or did participants engage in back-filling, how long did participants spend completing the EMA), (d) participant ratings of acceptability and feasibility, (e) participant reports of barriers and adverse events, (f) engagement with data dashboard (i.e., number of times the data visualization was accessed; aggregate anonymized data), and (g) participant ratings of data visualization use (qualitative and quantitative) collected using an investigator-created Follow-up Satisfaction and Feedback Questionnaire, included as Additional file 3. A small subset of participants will be invited (purposive sampling, to maximize the diversity of participant characteristics) to take part in a post-trial in-depth interview and questionnaire based on the User Experience Questionnaire (UEQ) scale [49], to review the utility, understandability, interestingness, aesthetics, and relatability of the visualizations and suggest improvements for the data visualizations; additional compensation will be provided for this subgroup.

#### Secondary outcomes

Relationships between daily measured variables will be assessed by examining the data collected through the EMA, as described above. This data will also be examined to determine preliminary differences between Part A and Part B.

#### Data management

As all participant-supplied data will be entered through REDCap or *CareTeam*, the data will be automatically entered into the electronic database by the participant. The use of digital data collection platforms means that only valid entries will be accepted (i.e., only values in range) and so that questionnaire scoring and reverse coding will occur automatically within REDCap, to reduce the change of human error in calculation.

### Statistical methods

#### Power calculation

A sample size of 50 was selected to be sufficient to determine a standard deviation for a sample size calculation for a larger clinical trial, as per published recommendations [50], and will also be sufficient to estimate a retention rate of 80% (95% CI = 69–91%).

### Data analysis

#### Primary outcomes

Descriptive statistics will be used to summarize participant feedback on the acceptability and feasibility of the EMA method of data collection and to examine participation and completion rates (e.g., what percentage of EMA time points yielded complete data, how many participants returned for the second period of data collection, timeliness of completion). Completion rates will be examined in relation to various demographic and pain-related factors, as well as in relation to the current condition (i.e., Part A vs Part B, as we hypothesize that there would be increased motivation and therefore better compliance during Part B as completing the EMA populates the data visualization). Content analysis will be used to examine common themes reported by participants in relation to feasibility, acceptability, barriers, and perceptions of the benefits or risks of participation [51].

#### Secondary outcomes

Multi-level modeling will be employed to account for the nested nature of the data and accommodate any missing data, examining patterns of association between variables (both concurrent and time-lagged) at the within- and between-participant level. Previous reports have indicated that a sample size of 50 is sufficient to conduct such analyses [13]. Specifically, we will examine whether stressful events (e.g., negatively perceived peer interactions) and avoidance at time 1 predict somatic and pain symptoms at time 2, moderated by anxiety. Qualitative content analysis will be used to summarize common themes reported by participants regarding the effectiveness of the visualization application based on their feedback provided in the follow-up questionnaire. Data gathered through the EMA over the length of the study will also be quantitatively analyzed to check for clinical improvement following Part A and Part B.

#### Interim analysis

No interim analysis will be conducted, aside from researchers regularly monitoring the responses to the free-text EMA questions and the feedback questionnaire question regarding emotional distress associated with study participation. Should there be sufficient reasons for concern this will be discussed with the Data Monitoring Committee (DMC), who will determine whether it is necessary to halt the trial.

#### Methods for additional analyses

Adolescent females are disproportionately affected by anxiety and somatic symptoms [52], and the negative mental health effects of pediatric chronic pain appear to be more common amongst treatment-seeking females [53]. Assigned sex and participant-identified gender will be measured using empirically supported, inclusive measures [42]. Sex and gender will be included as covariates in all primary analyses, and secondary analyses will investigate potential sex differences.

#### Methods in analysis to handle protocol non-adherence and any statistical methods to handle missing data

As this is a pilot feasibility study, information regarding protocol non-adherence and missing data will be considered important outcome data, and we will consider at what point did the participant drop out/not complete the data, and what were the reasons, if available, for the non-adherence or drop-out (e.g., technical issue, fatigue).

Preliminary analyses regarding the impact of the trial intervention will be conducted using an intention-to-treat analysis.

#### Plans to give access to the full protocol, participant level data, and statistical code

Full protocol and statistical code will be available along with the trial registration at osf.io/8scae. A de-identified dataset of the quantitative EMA data will be made publicly available (e.g., as supplementary materials to a publication or posting in a repository).

### Oversight and monitoring

#### Composition of the trial steering committee and data monitoring committee (DMC)

The trial steering committee, comprised of the authorship team, will report to the DMC, who will be comprised of a physician-scientist and psychologist.

#### Adverse event reporting and harms

The risks associated with participating in this study are low. Youth participants may find completing the questions 3 times a day during the EMA portion of the study an annoyance but are free to skip any data collection sessions they wish. If the prompt occurs during a time where it is inconvenient to complete, the participant may choose to ignore or complete the questions later. While completing the questionnaires, participants will be asked questions of a personal nature about thoughts, feelings, emotions, and physical/mental health experiences. Such questions may make participants feel uncomfortable, and they are not required to answer any questions they do not wish to answer. Information about crisis lines/mental health resources is provided in the consent/assent form to all participants, and all participants will already have access to mental health resources through their involvement with the Complex Pain Clinic. Additionally, at the end of each day of EMA data collection, youth participants will receive a prompt to download an application (*RootD*) designed to teach self-management strategies for managing symptoms, which they may find helpful. The research team will follow up with any youth who indicates that some aspect of the EMA participation was emotionally upsetting to them, to ensure we have conducted a thorough debrief of any study-related distress and referred to necessary resources. The research team will monitor the responses to free-text question fields of the questionnaires and EMA (e.g., “What are you most worried about happening today?”) at least twice a week during data collection to monitor for any actionable responses to those questions (e.g., suicidality or abuse disclosures), though none of the free-text questions is reasonably expected to provoke such responses. Study-related distress will be considered an adverse event and any instances of this, or any other adverse events, will be documented and discussed with the DMC and reported in any published findings.

#### Frequency and plans for auditing trial conduct

The DMC will be responsible for monitoring adherence to the trial protocol and raising any issues with respect to trial conduct. Therefore this process will be independent from the investigators and sponsor.

#### Plans for communicating important protocol amendments to relevant parties

Any important protocol amendments and their justification will be updated in the trial registration and will be submitted as an amendment for review by our local Research Ethics Board, and trial participants will be notified (only if the change impacts their current or past participation).

## Discussion

This patient-oriented proposal engages an at-risk population of youth in an innovative approach to measure and present longitudinal symptom data to support symptom self-management. Engagement with self-assessment, symptom appraisal, and management via a data visualization is a timely and novel approach that puts the youth in the “driver's seat” for self-managing their chronic pain and finding ways to improve daily function in ways that are meaningful to the individual in their daily lives. This project will serve as a pilot project to collect data on the feasibility and acceptability of this data collection and visualization method to administer to other high-risk populations and in preparation for a larger randomized controlled trial of this intervention. Importantly, feasibility and acceptability data will include both qualitative data of participant experience as well as objective measures of usage, which will provide a foundation for understanding the potential for this approach and how to improve the intervention and its implementation.

Barriers to equity, diversity, and inclusion are well-recognized determinants of care for people with chronic pain [54]. Overcoming barriers to care is a core objective of developing our real-world platform for data collection and treatment of chronic pain in children. Our proposal offers an approach to data collection and intervention that can be delivered remotely, requiring only access to a smartphone, to enhance the availability of this tool to youth who may otherwise have lacked access to research or clinical care for chronic pain. Recent work from our group suggests that access to smartphones and Internet/data are not frequently cited as a barrier to EMA research participation [38], and the present study will collect empiric data on the number of participants who decline participation due to lack of Internet/data access or require the use of a borrowed phone from the research team. As the present platform is delivered via a web browser rather than an application, there is no concern regarding phone storage serving as a barrier to participation or engagement. This data platform will ultimately improve access to clinical care for marginalized populations who typically lack access to participate in research and receive timely clinical care, support communication between patients and providers, and enable us to test the impact of an intervention that may allow children/youth and families reduce pain and costs (financial, social, and emotional) inherent to chronic childhood pain. Clinician feedback and integration with existing interventions will be examined in a subsequent study.

Gathering richer, more ecologically valid, and reliable data using EMA offers opportunities to integrate real-world data into clinical care that will reduce many of the barriers associated with collecting data from underrepresented and marginalized populations. Real-time analytics will assist researchers in developing AI (artificial intelligence)-based predictive modeling that should open new possibilities to individualize care, harness digital technology, and optimize health and well-being in everyday settings. While the benefits of using an EMA approach have already been described extensively, our hope is that pairing EMA with a data visualization intervention may harness the potential benefits of symptom monitoring and increase engagement with EMA data collection, as the user “benefits” from supplying more data and the process of data collection is not one-way.

This work will likely be relevant to other clinical populations, particularly those where behavior tracking, adherence, or self-management is of relevance, including most pediatric medical and psychiatric conditions. This intervention could be integrated within a CBT protocol, as the data visualization platform could be used for tracking symptom triggers and treatment progress, as well as using the data to challenge unhelpful cognitions.

### Dissemination plans

Our dissemination plan will include communication/dissemination of findings via peer-reviewed publications, websites, public health campaigns, social marketing, and workshops. We will provide the option for trial participants to opt-in to receiving a summary of the study findings at the conclusion of the trial. Our dissemination plan includes in-kind support from SKIP (Solutions for Kids in Pain) knowledge mobilization network, in partnership with the Canadian Mental Health Association to facilitate interactive dialogs with youth and their parents with lived experience, clinicians, and policy-makers. Our established connections with the SKIP, Canadian Pediatrics Society, Mental Health Commission of Canada, and BC Ministry of Health will enable rapid dissemination of findings to relevant stakeholders. If the results of a larger randomized clinical trial suggest the efficacy of this intervention, we will engage these same stakeholders in making this intervention available more broadly; as a low-resource low-barrier intervention, it may be ideally suited to be offered to patients who are waiting to be seen by a chronic pain clinic and to be integrated as part of a virtual care. We are not aware of any publication restrictions in place by our institution or funding bodies.

### Trial status

This trial is not yet open for recruitment. The study team is currently undergoing pilot-testing of the data visualization prototype with a small sample of youth with chronic pain, intended to obtain feedback to improve the design of the application prior to launching the trial. Note: Since the initial manuscript submission, recruitment commenced (May 2022).

## Supplementary Information

**Additional file 1.** A near-final draft of the sample visualizations (currently undergoing user testing).

**Additional file 2.** SPIRIT checklist.

**Additional file 3.** An investigator-created Follow-up Satisfaction and Feedback Questionnaire.

## Data Availability

Full protocol and statistical code will be available along with the trial registration at osf.io/8scae. A de-identified dataset of the quantitative EMA data will be made publicly available (e.g., as supplementary materials to a publication or posting in a repository).

## Abbreviations

AI: Artificial intelligence
CBT: Cognitive Behavioural Therapy
CONSORT: Consolidated Standards of Reporting Trials
CPS: Complex Pain Service
DMC: Data monitoring committee
EMA: Ecological momentary assessment
PROMIS: Patient-Reported Outcomes Measurement Information System®
SKIP: Solutions for Kids in Pain
SPIRIT: Standard Protocol Items: Recommendations for Interventional Trials

## Footnotes

1 Youth who took part in previous research to design and develop the visualization platform will not be eligible to take part in the present study, as their prior knowledge of the context and development of the visualizations may bias their experience.

2 Any participants who require this option will be offered an orientation to the phone with a member of the research team to ensure they are comfortable with the tasks required to complete the ratings and access the visualizations, prior to commencing the trial.
