# Supplementary figures and images for: Data visualization as an intervention for pediatric chronic pain: a pilot feasibility study protocol for a randomized controlled crossover trial
Source: Pilot Feasibility Stud. 2022 Oct 3;8:223. doi: 10.1186/s40814-022-01170-5 (PMC9527132; doi:10.1186/s40814-022-01170-5)

## Weeks of August 15 2021 to August 28 2021

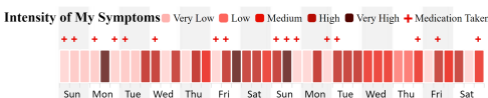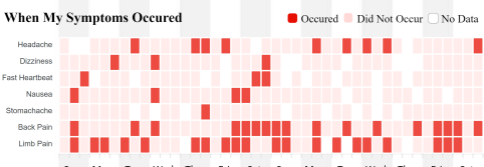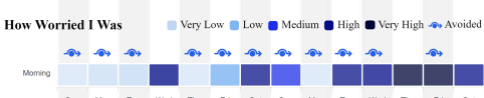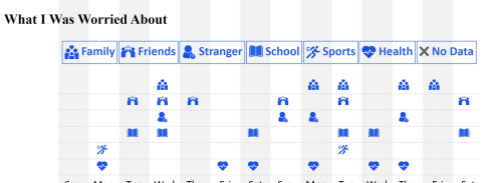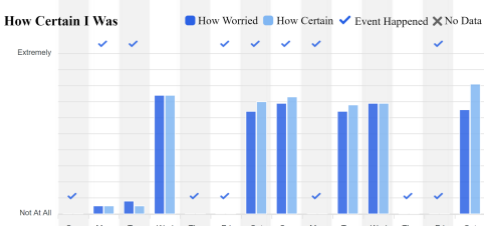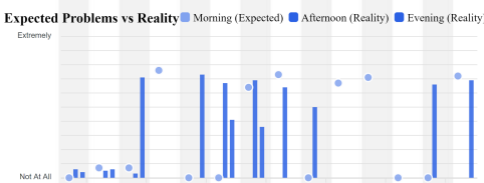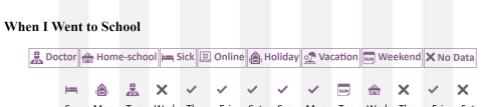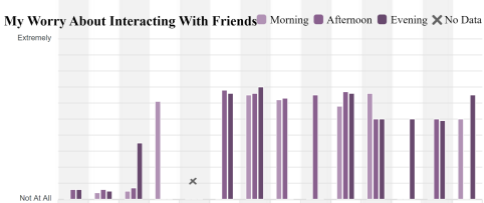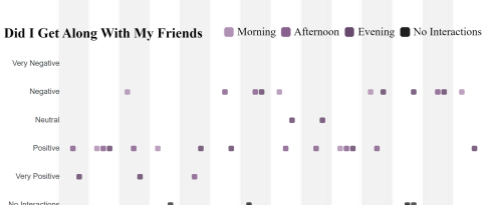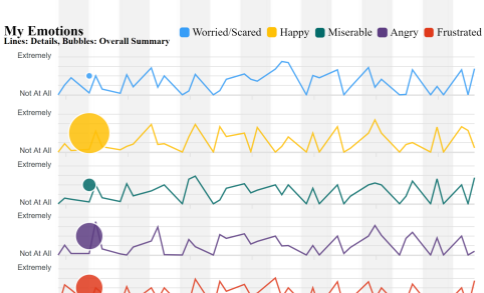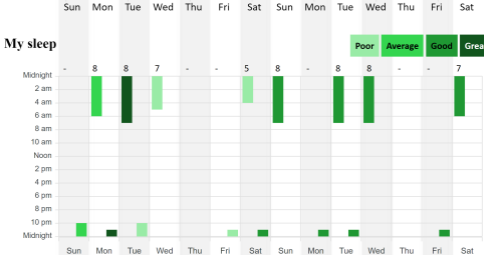

Supplement: Supplementary file 1 — Additional file 1. A near-final draft of the sample visualizations (currently undergoing user testing). [file 40814_2022_1170_MOESM1_ESM.pdf]
