# Supplementary material for: Data visualization as an intervention for pediatric chronic pain: a pilot feasibility study protocol for a randomized controlled crossover trial
Source: Pilot Feasibility Stud. 2022 Oct 3;8:223. doi: 10.1186/s40814-022-01170-5 (PMC9527132; doi:10.1186/s40814-022-01170-5)
Supplement: Supplementary file 3 — Additional file 3. An investigator-created Follow-up Satisfaction and Feedback Questionnaire. [file 40814_2022_1170_MOESM3_ESM.docx]

**Follow-up satisfaction and feedback questionnaire**

Thank you for completing the [first/second] week of this study! We have a few questions to ask you about your experience doing this study **this week**. Your answers will help us understand how you are finding the study and what we can do to make it better in the future. As a reminder, “EMA” means “Ecological Momentary Assessment”, which is the type of study you are doing where you answer a couple of questions 3 times a day on your smartphone for several days.

1. What did you like about participating in the EMA study? **[free text box]**
2. What did you dislike about participating in the EMA study? **[free text box]**
3. Did you have any problems in doing the EMA study? If so, please describe them here (e.g., technical difficulties, not having access to your smartphone when the text messages arrived, questions too hard to understand, forgot to answer): **[free text box]**
4. Did you find any part of participating in the EMA study emotionally distressing? **[choices: yes/no]** ****Note: Researcher will follow-up with any participant that indicates “yes” to ensure appropriate debriefing is conducted and any necessary mental health supports are in place***
5. How was it filling out questions 3 times a day? **[choices: 3 times a day was too much, 3 times a day was just right, 3 times a day was too few times]**
6. How did you find the number of questions asked each time? **[choices: too many questions, just right, too few questions]**
7. What did you think of the timing of the reminders? **[free text box]**
8. What did you do when you received the reminder if you were in the middle of doing something else? **[choices: stopped what I was doing and answered the questions, finished the task I was working on and did the questions after, did the questions later, other: {please specify}]**
9. Is the amount that you got paid to participate in this study enough for what we are asking you to do? **[choices: yes/no]**
10. What kinds of questions should we be answering with this kind of research? **[free text box]**
11. Was there anything that the EMA questions should have been asking about (e.g., different experiences, symptoms, emotions) that would have helped us better understand how your day was going? **[free text box]**
12. ***Asked only at the end of Phase B***: Did you look at the data visualization dashboard? (the place on the app where you could see charts and graphs of your data over time)
    1. [If yes:] What did you think of the data visualization dashboard? Tell us what you liked and didn’t like about it, and whether you found it useful. **[free text box]**
    2. [If no:] Why not? **[free text box]**
13. At the end of each day, you received a prompt to try an app for managing symptoms called *Rootd*. Did you try the app? **[choices: yes/no]**
    1. Did you find the *Rootd* program useful? **[choices: yes/no]**
    2. Would you recommend *Rootd* to a friend? **[choices: yes/no]**
14. During the past week of the study, did you use any of the following treatments for your pain?

- Over-the-counter medication (e.g., Advil, Tylenol)
- Prescription medication
- Physiotherapy
- Psychology
- Occupational therapy
- Naturopathic treatments
- Chiropractic treatments
- Massage therapy
- Acupuncture
- Hot/cold treatments
- Herbal remedies
- Counselling or other mental health treatments
- Other: _______________________

1. Do you have any other questions or feedback you would like to give us about participating in this study?
